# Correlation between gender-based violence and poor treatment outcomes among transgender women living with HIV in Brazil

**DOI:** 10.1186/s12889-024-18224-3

**Published:** 2024-03-13

**Authors:** Maria Amelia de Sousa Mascena Veras, Neia Prata Menezes, Adrienne Rain Mocello, Anna M. Leddy, Gustavo Santa Roza Saggese, Katia Cristina Bassichetto, Hailey J Gilmore, Paula Galdino Cardin de Carvalho, Luca Fasciolo Maschião, Torsten B. Neilands, Jae Sevelius, Sheri A. Lippman

**Affiliations:** 1 Department of Collective Health, Santa Casa School of Medical Sciences, São Paulo, Brazil; 2 Division of Prevention Science, Department of Medicine, University of California, San Francisco, San Francisco, USA

**Keywords:** Gender-based violence, HIV care, Transgender persons, Brazil, HIV, Medication adherence, Viral suppression

## Abstract

**Background:**

Transgender women are disproportionately affected by both HIV and gender-based violence (GBV), defined as physical, sexual, or emotional violence perpetrated against an individual based on their gender identity/expression. While a growing body of evidence demonstrates that GBV leads to poor HIV care and treatment outcomes among cisgender women, less research has examined this association among transgender women. We assessed the impact of lifetime experiences of GBV on subsequent retention in HIV care and laboratory confirmed viral suppression among a sample of transgender women living with HIV (TWH) in Brazil.

**Methods:**

A pilot trial of a peer navigation intervention to improve HIV care and treatment among TWH was conducted in São Paulo, Brazil between 2018 and 2019. TWH were recruited and randomized into the intervention or control arm and participated in a baseline and 9-month follow-up survey and ongoing extraction of clinical visit, prescribing, and laboratory data. Generalized linear model regressions with a Poisson distribution estimated the relative risk (RR) for the association of lifetime physical and sexual violence reported at baseline with treatment outcomes (retention in HIV care and viral suppression) at follow-up, adjusting for baseline sociodemographic characteristics.

**Results:**

A total of 113 TWH participated in the study. At baseline, median age was 30 years, and the prevalence of lifetime physical and sexual violence was 62% and 45%, respectively. At follow-up, 58% (*n* = 66/113) were retained in care and 35% (*n* = 40/113) had evidence of viral suppression. In adjusted models, lifetime physical violence was non-significantly associated with a 10% reduction in retention in care (aRR: 0.90, 95% CI: 0.67, 1.22) and a 31% reduction in viral suppression (aRR: 0.69; 95% CI: 0.43, 1.11). Lifetime sexual violence was non-significantly associated with a 28% reduction in retention in HIV care (aRR: 0.72, 95% CI: 0.52, 1.00) and significantly associated with a 56% reduction in viral suppression (aRR: 0.44; 95% CI: 0.24, 0.79).

**Conclusion:**

Our findings are among the first to demonstrate that lifetime experiences with physical and sexual violence are associated with poor HIV outcomes over time among transgender women. Interventions seeking to improve HIV treatment outcomes should assess and address experiences of GBV among this population.

**Trial registration:**

ClinicalTrials.gov Identifier: NCT03525340.

**Supplementary Information:**

The online version contains supplementary material available at 10.1186/s12889-024-18224-3.

## Background

Brazilian transgender women experience great disparities in HIV [1], with 55 times higher odds of HIV infection than the general population [2]. Among transgender women living with HIV (TWH) in Brazil, HIV care engagement falls short of the UNAIDS 95-95-95 targets. A recent study from São Paulo found that 81% of TWH in 2016–2017 knew their HIV status; of those, 77% had been linked to care [3]. Another study from Rio de Janeiro found that only 35% of TWH in 2015–2016 had an undetectable viral load [4].

The recent UNAIDS targets also include a new goal: that less than10% of members of at-risk communities experience gender inequality and violence [5]. A recent meta-analysis found that transgender individuals had 2.2 times the risk of experiencing physical gender-based violence (GBV), compared to cisgender individuals, and 2.5 times the risk of sexual GBV [6]. Transgender women in Brazil experience some of the highest prevalences of GBV in the world [7, 8]. GBV against transgender women includes physical, sexual, and emotional violence due to stigmatization of gender nonconformity and gender expression/identity [9–11]. In its most extreme form, GBV leads to homicide [8, 12, 13].

Despite Brazil’s progressive legislation supporting lesbian, gay, bisexual, transgender, and intersex (LGBTI) populations, including constitutional sanctioning of same-sex marriage and the right to change one’s legal name according to gender identity, a staggering 29% of all transgender murders globally occur in Brazil [14].

Among cisgender women, history of GBV is a well-established determinant of HIV risk [15–18], and a growing body of research demonstrates its association with suboptimal HIV care and treatment [19, 20]. However, many of these studies were limited methodologically by small sample size, use of self-reported data, and/or cross-sectional study designs [19]. Few studies have examined the impact of GBV on HIV care and treatment outcomes among TWH [21, 22], with no studies to date in Brazil, home to a vibrant transgender population. We examined the impact of GBV on retention in HIV care and viral suppression among Brazilian TWH in São Paulo.

## Methods

### Study setting and procedures

We conducted a secondary analysis of data from Trans Amigas, a pilot study of a peer navigation intervention to improve engagement in HIV care among TWH in São Paulo, Brazil [23]. Participants were recruited between May to November 2018 from our collaborating clinic, the Centro de Referência e Treinamento (CRT) DST/AIDS (São Paulo State HIV Reference and Training Center), through community-based outreach, and from among participants from the Transnational cohort study who had tested positive for HIV [24]. Full details of the study procedures have been previously described [23].

Trans Amigas participants were 18 years or older, assigned ‘male’ at birth but currently identified as female or transgender, and either resided, worked, or studied in São Paulo. We enrolled participants who either had a recent HIV diagnosis (prior 12 months) and were willing to enroll in HIV care at CRT, or who were already enrolled at CRT for HIV care and were interested in improving their retention in care. We were interested in assessing the impact of navigation among TWH who had not yet formed retention in care habits (recently diagnosed) and those who required more support for retention and adherence. While we could not confirm treatment adherence prior to enrollment, as we did not have access to clinic records until consent was signed, we did not enroll those in care at CRT who reported being fully adherent with no need of navigation support. Additionally, those preferring to receive care elsewhere were not enrolled, as access to clinical records and stationing personnel at additional clinics was beyond the scope of the study. Participants were enrolled following eligibility confirmation and informed consent, in accordance with the Declaration of Helsinki. Ethical approval was obtained from the Institutional Review Board from the University of California, San Francisco, the Comitê de Ética em Pesquisa at the CRT DST/AIDS, the Santa Casa de São Paulo, and the Brazilian National Ethics Committee, CONEP.

A total of 113 TWH participated in our study, with 75 randomized to the intervention and 38 to the control condition. Overall, 38 had a recent HIV diagnosis and 71 had a prior history of HIV care at CRT. We were unable to verify clinical date of HIV diagnosis for four participants. Participants randomized to the control were referred into our partnering clinic, a transgender-friendly HIV clinic located within a local primary care facility already providing services to transgender women. Intervention participants were paired with a peer navigator (PN), who was a TWH engaged in HIV care with extensive empowerment-based training.

All participants were followed for nine months, with surveys at enrollment and nine months. HIV clinical outcome data were extracted from three data sources: (a) clinical visits from an electronic clinical charting system at the study clinic, (b) medication dispensing history from the national medications dispensing system, and (c) viral load results from the national laboratory tracking system.The Brazilian national reporting system for medications and laboratory results is the same across all government services and facilitates the locating of records for patients who access services at multiple facilities. At enrollment, we asked participants for any legal and social names used and searched for all names in all databases.

### Measures

#### Outcome

The primary outcome was retention in HIV care at follow-up, defined as having a confirmed HIV clinical visit or antiretroviral therapy (ART) medication pick-up around nine months from enrollment (using the window of 7 ½ to 10 ½ months given the three-month treatment interval) [23]. The secondary outcome was viral suppression (viral load < 1,000 copies/mL) at follow-up.

#### Exposures

Key exposures were self-reported lifetime experience of physical and sexual violence (GBV) collected at baseline. Participants were asked whether they had experienced physical or sexual violence due to their gender identity or presentation: “Being a transgender person, have you ever had to deal with: A) Physical assault (yes/no) or B) Sexual violence (yes/no)”. The study utilized lifetime exposure given the documented association between any history of GBV and care engagement in HIV-positive populations [25]. Of note, although participants were asked about verbal violence—“Being a transgender person, have you ever had to deal with verbal aggression/slurs/humiliation?”—we chose not to explore verbal aggression in assessing impacts on HIV outcomes as nearly every participant (92%) reported experiencing verbal aggression.

#### Covariates

Baseline covariates included age, education (completed secondary school vs. not), monthly income, hazardous alcohol use (vs. none), any drug use in the past six months (vs. none) and intervention arm (vs. control). Monthly income was dichotomized at 800 BRL (∼ USD 5.50/day for 30 days), which is equivalent to the lowest quintile of income in Brazil [26]. Hazardous alcohol use was assessed by the three item Alcohol Use Disorders Identification Test–Consumption (AUDIT-C) [27]. Participants rated the frequency and quantity of alcohol they consumed using a 4-point Likert scale. Scores were combined across items to obtain the overall score (range 0–12) and dichotomized using the cutoff of 4 for hazardous drinking [27]. Participants also provided information on housing stability (stable vs. unstable housing [i.e., homeless, shelter]), lifetime experience with incarceration, and social support, as measured using the Social Provisions Scale [28].

#### Analysis

 Based on our thorough extraction of the national clinical databases,those missing HIV care and viral load data in their medical records at follow-up were assumed for our primary analysesnot to be retained in care (*n* = 16; 14%) or virally suppressed (*n* = 65; 57%).Generalized linear model (GLM) regressions with Poisson distributions and robust variance estimates were used to estimate the relative risk (RR) of retention in care and viral suppression by lifetime physical and sexual violence, separately, adjusting for covariates. All models adjusted for log of age (years), intervention arm, and education *a priori.* Additional covariates were considered based on statistically significant association with the outcomes of interest (*p* < 0.20). We additionally considered effect measure modification by race (white vs. person of color [POC]), collapsing individuals identifying as Asian, Black, Mixed/Parda, or Indigenous into a single category due to small cell sizes. Finally, we conducted sensitivity analyses to account for the assumption that those missing in national databases were not retained or virally suppressed. We first restricted our analytical sample to participants with complete outcome data (*n* = 97 and *n* = 48 with treatment and viral load data, respectively). We then conducted an additional analysis for the viral load outcome, whereby participants with missing viral load data who also did not pick up their medication were classified as not virally suppressed (n=27); those missing viral load information but who had medication pick up documented were not included. This was a more conservative assumption than assuming that all those with missing viral load data were not virally suppressed. We also disaggregated analyses by enrollment status (recent HIV diagnosis vs. previously in HIV care) to assess differences in findings by prior history of HIV care.

## Results

The median age at baseline was 30 years, and 30% of participants had earnings in the lowest quintile of income in Brazil **(**Table 1**)**. Overall, 35% (*n* = 40) of participants completed secondary school or more, 28% (*n* = 32) had incomplete secondary school, and 36% (*n* = 41) had primary school or less.

Prevalence of lifetime physical and sexual violence was 62% and 45%, respectively; 40% of participants reported experiencing both physical and sexual violence in their lifetime. At follow-up, 58% (*n* = 66/113) were retained in HIV care and 35% (*n* = 40/113) had confirmed viral suppression.

In multivariable analyses, compared to those who never experienced physical violence, TWH who experienced lifetime physical violence were 10% less likely to be retained in HIV care (adjusted relative ratio [aRR]: 0.90, 95% CI: 0.67, 1.22) and 31% less likely to be virally suppressed (aRR: 0.69, 95% CI: 0.43, 1.11), however findings were not statistically significant (Table 2). Compared to those who never experienced lifetime sexual violence, TWH who experienced lifetime sexual violence were 28% less likely to be retained in care (aRR: 0.72, 95% CI: 0.52, 1.00) and 56% less likely to be virally suppressed (aRR: 0.44, 95% CI: 0.24, 0.79). These findings signaled a statistically significant relationship between lifetime experiences of sexual violence and HIV clinical outcomes. We did not detect significant effect measure modification by race (Supplemental Tables 1 & 2). However, there is suggestive evidence that the relationship between sexual violence and retention in care was potentially stronger among women of color. We did not pursue additional stratified analyses due to our small sample size. Sensitivity analyses indicate that assumptions regarding missing HIV care data did not appreciably alter findings from primary analyses, however, associations regarding missing viral load data were attenuated when considering complete cases only (Supplemental Table 3). After accounting for no indication of medication pick up, viral suppression results were in line with the primary analysis (Supplemental Table 4). After disaggregating analyses by enrollment status, we found no appreciable differences in findings (Supplemental Tables 5a-b).

**Table 1:** Baseline characteristics of transgender women living with HIV enrolled in the Trans Amigas cohort (*n* = 113)

Baseline Characteristics	n (%)
Median age (Interquartile Range [IQR])	30 (25–39)
Earnings in the lowest quintile (≤ 800 BRL per month)	34 (30.1)
Completed secondary school	40 (35.4)
Hazardous alcohol consumption	69 (61.1)
Race / Ethnicity	
White	36 (31.9)
Black	13 (11.5)
Mixed race (parda)	54 (47.8)
Asian (amarela)	2 (1.8)
Indigenous	8 (7.1)
Drug use in the past six months	75 (66.4)
Unstable housing	18 (15.9)
Lifetime incarceration	32 (28.3)
Median social support score (IQR)	18 (14–23)
Intervention arm (vs. control)	75 (66.4)
Lifetime physical violence	70 (61.9)
Lifetime sexual violence	51 (45.1)
**Summary lifetime violence**	
No physical or sexual violence	37 (32.7)
Physical or sexual violence	31 (27.4)
Both physical and sexual violence	45 (39.8)

**Table 2:** Adjusted relative risk (RR) of lifetime physical and sexual violence on retention in care and viral suppression among transgender women living with HIV in Brazil (*n* = 113) ^*^

	A. Retention in Care		B. Viral Suppression	
	RR (95% CI)	aRR (95% CI)	RR (95% CI)	aRR (95% CI)
**Lifetime Physical Violence**				
No	*Ref.*	*Ref.*	*Ref.*	*Ref.*
Yes	0.89 (0.65, 1.21)	0.90 (0.67, 1.22)	0.61 (0.38, 1.00)	0.69 (0.43, 1.11)
**Lifetime Sexual Violence**				
No	*Ref.*	*Ref.*	*Ref.*	*Ref.*
Yes	0.74 (0.53, 1.03)	0.72 (0.52, 1.00)	0.40 (0.22, 0.75)	0.44 (0.24, 0.79)

* Multivariable models adjust for log age (years), intervention arm, completed secondary/high school education, income (dichotomized at <$800 reais per month), perceived social support, housing stability, and hazardous alcohol use (dichotomized using AUDIT-C Scale: none/moderate consumption [scores < 3] vs. hazardous consumption [scores ≥ 3]. RR = unadjusted relative risk; aRR = adjusted relative risk; CI = confidence interval

## Discussion

We sought to examine the association of GBV on retention in HIV care and viral suppression among Brazilian transgender women living with HIV in São Paulo. We found evidence of a relationship between reporting a history of sexual violence and poor HIV care engagement and viral suppression, and a non-significant association between experienced physical violence and unsuppressed viral load. We also noted extremely elevated reporting of lifetime physical and sexual violence among the transgender women in our cohort.

Our findings corroborate reports of an extremely high prevalence of lifetime physical and sexual violence among transgender women in Brazil. A recent study of 763 transgender women in São Paulo found that prevalence of lifetime physical violence and sexual violence were 62% and 20%, respectively [29]. TWH in the present study reported similar prevalence of physical violence (62%) and even higher levels of sexual violence (45%). TWH may have greater risk of experiencing sexual violence compared to transgender women who are not living with HIV [30]; however, this needs further exploration.

Our results support evidence documenting lower prevalence of engagement in HIV care and viral suppression among cisgender women who experienced GBV [20, 31–35]. In addition to aligning with the literature on the relationship between GBV and engagement in HIV care among cisgender women, our findings build upon cross-sectional studies documenting an association between GBV and viral load among transgender women in the United States (US). One US study found that recent violence (defined as reporting being “abused, threatened or the victim of violence” in the past 30 days) was cross-sectionally associated with increased odds of having a detectable viral load [21]. Another study found that recent physical assault and intimate partner violence (IPV) were inversely associated with self-reported viral suppression among Black/African American transgender women in the US [22].

GBV has been associated with suboptimal ART adherence [31, 32, 36–40], which can result in drug-resistant HIV strains and decreased viral suppression [41]. Additionally, GBV has been demonstrated to contribute to HIV disease progression and the presence of opportunistic infections in women living with HIV [42], which may contribute to lack of retention in HIV care and viral suppression. Mediation analysis could help tease apart the direct and indirect effects in these relationships. However, intervening on GBV would address the most upstream cause of the observed adverse HIV outcomes.

While we are unaware of prior quantitative studies documenting a relationship between GBV and retention in HIV care among TWH, qualitative research suggests that past experiences of violence, and associated trauma, prevent engagement in HIV care among this population [43–45]. Other research has demonstrated that GBV is associated with TGW healthcare avoidance more broadly in South America [46] and that GBV is negatively associated with PrEP adherence among transgender women in the US [47].

To our knowledge, this is among the first studies to demonstrate that lifetime experiences of sexual violence are associated with subsequent reduced likelihood of retention in HIV care and laboratory confirmed viral suppression among TWH. And while we did not find a significant relationship between lifetime physical violence and treatment outcomes in this small sample, the relationships are in the expected direction, as experiences of physical violence have been found to impact self-care [20, 48, 49].

Additionally, 88.2% of women reporting sexual violence in our cohort also reported physical violence, while fewer (64.3%) reporting physical violence also reported sexual violence. Past research has shown that transgender women experience multiple forms of violence and discrimination throughout their life course [50, 51]. Polyvictimization has been associated with HIV outcomes in cisgender populations [52] and among gender minority groups has been linked to higher prevalence of anxiety, depression, drug use, and suicidality [53]. It is possible that experiences of sexual violence could lead to worse HIV outcomes through a path of poor mental health. GBV is associated with post-traumatic stress disorder (PTSD) and depression [50], which are, in turn, associated with reduced engagement in HIV care, ART adherence and viral suppression [54–57]. Future longitudinal research with sufficient power should unpack the potential mechanisms linking sexual violence to poor HIV treatment outcomes among TWH and whether the relationship between physical violence and HIV care behaviors is indeed smaller than the relationship we found with sexual violence. We also recommend further research into the potentially stronger relationship between sexual violence and HIV care retention among women of color.

Finally, it is important to stress that this study was conducted in the context of some of the most progressive policies for LGBTI populations in the world. That transgender women are experiencing such great disparities in prevalence of violence, HIV infection, and treatment outcomes demonstrates that progressive policies are not sufficient. Our findings support past research that has highlighted the disconnect between Brazilian laws and policies designed to protect LGBTI rights and guarantee universal healthcare access, and the true access these populations experience [58, 59]. These results indicate the urgent need for programs designed to prevent GBV and improve HIV care engagement among Brazilian TWH, including programs that improve the implementation of existing policies designed to support their health and human rights, and accountability should programs not be implemented in accordance with existing policies.

### Limitations

This was a pilot study with a small sample size and findings are not generalizable outside of the study clinic and area. We did not collect detailed information about different forms of violence (e.g., being slapped, hit, beaten, etc.), timing of violence (e.g. recency and frequency), or violence perpetrator (e.g., sexual partner, stranger). By opting to use a lifetime measure, we cannot make inferences about the association of recent experiences of GBV with HIV clinical outcomes. Furthermore, violence perpetrated by an intimate partner or family member may be more likely to recur, compared to violence from a stranger, and have a more prolonged impact on HIV outcomes. A study in São Paulo using data from 2012 to 2014 found that cisgender women living with HIV, compared to HIV-negative cisgender women, were more likely to experience: lifetime physical violence (44% and 32%, respectively); repeated physical violence (31% vs. 19%); and physical violence perpetrated by an intimate partner (66% vs. 56%) [30]. Although not captured in our study, this information may have yielded a more nuanced understanding of the impact of GBV on HIV outcomes and provided further insight into potential targets for intervention.

Measures of violence victimization are self-reported and may be prone to social desirability of reporting bias. New literature [60] suggests that recent experiences of GBV may be more predictive of HIV care engagement compared to lifetime history; our use of lifetime history may have attenuated our ability to measure the true relationship between experiences of GBV and engagement in HIV care. Moreover, tools are now available to measure transgender-specific forms of violence, including having hormones destroyed by a partner or being pressured to not pursue gender-affirming care [61]. Partner-controlling tactics such as destruction of medication and prevention of access to healthcare services could play a direct role in viral suppression and retention in HIV care. Future studies should utilize measures that clearly define different violent behaviors to facilitate meaningful comparisons across settings [62]. Furthermore, we lacked follow-up viral load data for 57% of the sample. It is possible that the more pronounced association with viral suppression reflects a mix of both the negative impact of sexual abuse on transgender women’s ability to engage in care (and thus access viral load testing) and a deterrent to medication adherence. Our finding that sexual violence was associated with less retention in care suggests that this may be the case; however, future research should confirm and unpack these relationships with larger sample sizes.

Other clinical variables frequently correlated with retention in HIV treatment were not included. We also excluded factors like recent incarceration or recent drug use from multivariable analyses, given the lack of association with our outcomes of interest in this sample. Considering the extant literature suggesting incarceration and drug use as possible confounders of the relationship between GBV and HIV clinical outcomes, it is possible that our sample size was too small to detect these associations. Variables unmeasured in our study, including whether providers delivered confidential, supportive, and transgender-friendly care, could further confound the observed relationships between GBV and HIV outcomes, as prior research has noted that victims of violence are particularly sensitive to care context [63]. Finally, although we did not detect significant or interpretable differences in associations by race, our study was likely insufficiently powered to assess effect modification by race. Other studies have indicated racial disparities in HIV retention and viral suppression among transgender women [64].

## Conclusion

Our results reiterate the extremely high prevalence of GBV among transgender women in Brazil and demonstrate that lifetime experiences of sexual violence are predictive of reduced likelihood of retention in HIV care and viral suppression among TWH. These findings point to an urgent need to protect the human rights of transgender women and for targeted programming for TWH to facilitate access to safe, supportive, and quality health care, devoid of stigma and discrimination. Interventions to improve HIV care engagement may include addressing transphobia among healthcare providers as well as “know your rights” campaigns, whereby marginalized groups learn about their rights and are trained on mobilizing approaches to advocate for their rights in partnership with sympathetic stakeholders [11, 65, 66]. It is also important to continue to support the ongoing grassroot efforts to promote health and human rights of transgender women in Brazil [66].

### Electronic supplementary material

Below is the link to the electronic supplementary material.

Supplementary Material 1

## Data Availability

The datasets generated and analyzed during the current study are available from the corresponding author upon reasonable request.
