# Supplementary material for: Correlation between gender-based violence and poor treatment outcomes among transgender women living with HIV in Brazil
Source: BMC Public Health. 2024 Mar 13;24:791. doi: 10.1186/s12889-024-18224-3 (PMC10938823; doi:10.1186/s12889-024-18224-3)
Supplement: Supplementary file 1 — Supplementary Material 1 [file 12889_2024_18224_MOESM1_ESM.docx]

Supplemental Tables

Table of Contents

[Table S1. Multivariable associations between lifetime physical and sexual violence and retention in HIV care among transgender women in Brazil, stratified by race ^*^ 2](#_Toc156171410)

[Table S2. Multivariable associations between lifetime physical and sexual violence and viral load suppression among transgender women in Brazil, stratified by race ^*^ 3](#_Toc156171411)

[Table S3. Adjusted relative risk (RR) of lifetime physical and sexual violence on retention in care and viral suppression among transgender women living with HIV in Brazil: Sensitivity analysis using only complete cases ^*^ 4](#_Toc156171412)

[Table S4. Adjusted relative risk (RR) of lifetime physical and sexual violence on viral suppression among transgender women living with HIV in Brazil: Sensitivity analysis limiting analytic sample to participants with viral load data and/or no indication of medication pick-up ^*^ 5](#_Toc156171413)

[Table S5a-b. Adjusted relative risk (RR) of lifetime physical and sexual violence on (a) retention in care and (b) viral suppression among transgender women living with HIV in Brazil: Sensitivity analysis disaggregating findings by enrollment status (recent diagnosis vs. previously in care) ^*^ 6](#_Toc156171414)

## **Table S1.** Multivariable associations between lifetime physical and sexual violence and retention in HIV care among transgender women in Brazil, stratified by race ^*^

|  | **White population** | **People of Color** |  |
| --- | --- | --- | --- |
|  | aRR (95% CI) | aRR (95% CI) | Interaction p-value |
| **Lifetime Physical Violence** |  |  |  |
| No | *Ref.* | *Ref.* |  |
| Yes | 0.77 (0.48, 1.24) | 0.95 (0.63, 1.44) | 0.52 |
| **Lifetime Sexual Violence** |  |  |  |
| No | *Ref.* | *Ref.* |  |
| Yes | 1.03 (0.63, 1.67) | 0.62 (0.41, 0.93) | 0.12 |

^*^ N=36 for white population and N=77 for POC. Multivariable models adjust for log age (years), intervention arm, completed secondary/high school education, income (dichotomized at $800 reais per month), perceived social support, housing stability, and hazardous alcohol use (dichotomized using AUDIT-C Scale: none/moderate consumption [scores <3)] vs. hazardous consumption [scores ≥3]). aRR = adjusted relative risk; CI = confidence interval; POC = participant self-identified as person of color (i.e., Black, Asian, Mixed/Parda, or Indigenous).

## **Table S2.** Multivariable associations between lifetime physical and sexual violence and viral load suppression among transgender women in Brazil, stratified by race ^*^

|  | **White population** | **People of Color** |  |
| --- | --- | --- | --- |
|  | aRR (95% CI) | aRR (95% CI) | Interaction p-value |
| **Lifetime Physical Violence** |  |  |  |
| No | *Ref.* | *Ref.* |  |
| Yes | 0.49 (0.23, 1.05) | 0.83 (0.44, 1.58) | 0.30 |
| **Lifetime Sexual Violence** |  |  |  |
| No | *Ref.* | *Ref.* |  |
| Yes | 0.19 (0.03, 1.20) | 0.51 (0.26, 1.00) | 0.33 |

^*^ N=36 for white population and N=77 for POC. Multivariable models adjust for log age (years), intervention arm, completed secondary/high school education, income (dichotomized at $800 reais per month), perceived social support, housing stability, and hazardous alcohol use (dichotomized using AUDIT-C Scale: none/moderate consumption [scores <3)] vs. hazardous consumption [scores ≥3]). aRR = adjusted relative risk; CI = confidence interval; POC = participant self-identified as person of color (i.e., Black, Asian, Mixed/Parda, or Indigenous).

## **Table S3**. Adjusted relative risk (RR) of lifetime physical and sexual violence on retention in care and viral suppression among transgender women living with HIV in Brazil: Sensitivity analysis using only complete cases ^*^

|  | **A. Retention in Care**  N = 97 | | **B. Viral Suppression**  N = 48 | |
| --- | --- | --- | --- | --- |
|  | RR (95% CI) | aRR (95% CI) | RR (95% CI) | aRR (95% CI) |
| **Lifetime Physical Violence** |  |  |  |  |
| No | *Ref.* | *Ref.* | *Ref.* | *Ref.* |
| Yes | 0.85 (0.65, 1.11) | 0.82 (0.62, 1.08) | 0.78 (0.61, 0.99) | 0.82 (0.66, 1.01) |
| **Lifetime Sexual Violence** |  |  |  |  |
| No | *Ref.* | *Ref.* | *Ref.* | *Ref.* |
| Yes | 0.77 (1.03, 0.76) | 0.76 (0.57, 1.01) | 0.73 (0.50, 1.07) | 0.76 (0.55, 1.04) |

^*^ Multivariable models adjust for log age (years), intervention arm, completed secondary/high school education, income (dichotomized at $800 reais per month), perceived social support, housing stability, and hazardous alcohol use (dichotomized using AUDIT-C Scale: none/moderate consumption [scores <3] vs. hazardous consumption [scores ≥3]). RR = relative risk; aRR = adjusted risk ratio; CI = confidence interval.

## **Table S4**. Adjusted relative risk (RR) of lifetime physical and sexual violence on viral suppression among transgender women living with HIV in Brazil: Sensitivity analysis limiting analytic sample to participants with viral load data and/or no indication of medication pick-up ^*^

|  | **Viral suppression**  N = 75 |
| --- | --- |
|  | aRR (95% CI) |
| **Lifetime Physical Violence** |  |
| No | *Ref.* |
| Yes | 0.63 (0.43, 0.92) |
| **Lifetime Sexual Violence** |  |
| No | *Ref.* |
| Yes | 0.48 (0.29, 0.82) |

* We assumed that participants with missing viral load data who had no indication of medication pick up were not virally suppressed. This added an additional 27 participants to our analysis who were not virally suppressed (total = 35). Multivariable models adjust for log age (years), intervention arm, completed secondary/high school education, income (dichotomized at $800 reais per month), perceived social support, housing stability, and hazardous alcohol use (dichotomized using AUDIT-C Scale: none/moderate consumption [scores <3)] vs. hazardous consumption [scores ≥3]). aRR = adjusted relative risk; CI = confidence interval.

## **Table S5a-b**. Adjusted relative risk (RR) of lifetime physical and sexual violence on (a) retention in care and (b) viral suppression among transgender women living with HIV in Brazil: Sensitivity analysis disaggregating findings by enrollment status (recent diagnosis vs. previously in care) ^*^

1. **Retention in care**

|  | **Recent HIV diagnosis**  N = 38 | **Previously in HIV care**  N = 71 |
| --- | --- | --- |
|  | aRR (95% CI) | aRR (95% CI) |
| **Lifetime Physical Violence** |  |  |
| No | *Ref.* | *Ref.* |
| Yes | 0.91 (0.64, 1.30) | 0.85 (0.48, 1.49) |
| **Lifetime Sexual Violence** |  |  |
| No | *Ref.* | *Ref.* |
| Yes | 0.77 (0.52, 1.15) | 0.78 (0.45, 1.34) |

1. **Viral suppression**

|  | **Recent HIV diagnosis**  N = 38 | **Previously in HIV care**  N = 71 |
| --- | --- | --- |
|  | aRR (95% CI) | aRR (95% CI) |
| **Lifetime Physical Violence** |  |  |
| No | *Ref.* | *Ref.* |
| Yes | 0.78 (0.45, 1.36) | 0.58 (0.20, 1.68) |
| **Lifetime Sexual Violence** |  |  |
| No | *Ref.* | *Ref.* |
| Yes | 0.46 (0.23, 0.89) | 0.48 (0.14, 1.70) |

***** Analyses exclude 4 participants for whom we were unable to obtain data regarding date of HIV diagnosis. Multivariable models adjust for log age (years), intervention arm, completed secondary/high school education, income (dichotomized at $800 reais per month), perceived social support, housing stability, and hazardous alcohol use (dichotomized using AUDIT-C Scale: none/moderate consumption [scores <3)] vs. hazardous consumption [scores ≥3]). aRR = adjusted relative risk; CI = confidence interval.
